# Cardioprotective effect of carvedilol: inhibition of apoptosis in H9c2 cardiomyocytes via the TLR4/NF-κB pathway following ischemia/reperfusion injury

**DOI:** 10.3892/etm.2014.1863

**Published:** 2014-07-24

**Authors:** YONG ZHAO, YAN XU, JIANHUA ZHANG, TINGTING JI

**Affiliations:** 1 Department of Emergency, The First Affiliated Hospital of Anhui Medical University, Hefei, Anhui 230032, P.R. China; 2 Department of Cardiovascular Medicine, The First Affiliated Hospital of Anhui Medical University, Hefei, Anhui 230032, P.R. China

**Keywords:** carvedilol, H9c2, apoptosis, ischemia/reperfusion, toll-like receptor 4/nuclear factor κ-light-chain-enhancer of activated B cells

## Abstract

Carvedilol is a non-selective β-blocker used in the treatment of cardiovascular disease, including myocardial ischemia. The aim of the present study was to investigate the molecular mechanisms underlying the effects of carvedilol on simulated ischemia/reperfusion (SI/R)-induced cardiomyocyte apoptosis *in vitro*. H9c2 cardiomyocytes were incubated with either a vehicle or an ischemic buffer during hypoxia followed by reoxygenation with or without carvedilol. In two additional groups, toll-like receptor 4 (TLR4) and nuclear factor κ-light-chain-enhancer of activated B cells (NF-κB) were inhibited by a TLR4 antibody and pyrrolidine dithiocarbamate, respectively. The results revealed that carvedilol markedly decreased SI/R-induced apoptosis in a concentration-dependent manner, as demonstrated by flow-cytometric analysis. This effect was shown to be associated with an increase in the B-cell lymphoma 2 (Bcl-2)/Bcl-2-associated X (Bax) protein ratio and concurrent reductions in the expression levels of TLR4 and NF-κB. These results suggest that carvedilol provides significant cardioprotection against SI/R-induced injury in H9c2 cardiomyocytes, an effect likely to be mediated through the TLR4/NF-κB signaling pathway.

## Introduction

Ischemia/reperfusion (I/R) injury is a common occurrence during thrombotic disease. The effects of reperfusion are typically beneficial but often lead to an inflammatory response that causes further damage to viable tissue around the infarct, a result thought to be associated with increased apoptosis, especially in cardiomyocytes (1). This form of damage has been demonstrated to lead to a loss in cardiomyocytes and an increased infarct size (2).

There are a number of signaling pathways that participate in cardiomyocyte apoptosis; however, the toll-like receptor 4 (TLR4)/nuclear factor κ-light-chain-enhancer of activated B cells (NF-κB) signal pathway is known to play one of the key roles in I/R injury (3,4) and has been observed to become markedly upregulated in failing and ischemic myocardium (5). Conversely, TLR4 deficiency increases the survival rate of cardiomyocytes following myocardial ischemia via an apoptosis-mediated effect (6). As such, one of the primary goals of therapeutic intervention during I/R injury is to increase myocardial protection by inhibiting apoptosis.

Carvedilol is a non-selective β-blocker initially used in the treatment of hypertension and angina (7). It has been shown to reduce the risk of hospitalization and the mortality rate in patients with severe chronic heart failure (CHF) (8). In a number of clinical trials (9,10) carvedilol has been demonstrated to exert a beneficial effect on ventricular remodeling and in preserving the ejection fraction, as compared with other blockers. These effects are considered to be mediated through multiple mechanisms, including the inhibition of cardiomyocyte apoptosis (11,12). However, the association between the effect of carvedilol on anti-apoptosis and the TLR4 signaling pathway is not yet known.

Therefore, the present study investigated the possibility that the TLR4 signaling pathway is involved in the anti-apoptotic effects of carvedilol. The effects of carvedilol were investigated in the H9c2 cardiomyocyte cell line following simulated I/R (SI/R) and the apoptosis rate was subsequently observed in order to explore the possible internal association between the effects of carvedilol and the TLR4 signaling pathway.

## Materials and methods

### Cell culture

The rat H9c2 cardiomyocyte cell line was obtained from the American Type Culture Collection (ATCC, Manassas, VA, USA). The cells were maintained in Dulbecco’s modified Eagle’s medium (DMEM, Hyclone, Logan, UT, USA) supplemented with 10% fetal calf serum at 37.1°C under CO_2_ incubation. The medium was replaced every 2–3 days and the cells were subcultured or subjected to experimental procedures at 80–90% confluence.

### SI/R injury model

Cardiomyocytes were randomly divided into seven groups: i) control; ii) SI/R; iii) carvedilol (1 μM); iv) carvedilol (5 μM); v) carvedilol (10 μM); vi) TLR4 inhibitor (20 μg/ml; an anti-TLR4 blocking antibody; CST Technologies, Boston, MA, USA) and; vii) pyrrolidine dithiocarbamate (100 μmol/l PDTC; NF-κB inhibitor) groups. In the control group, H9c2 cardiomyocytes were cultured under normal conditions in 5% CO_2_ incubation. SI/R experiments were performed according to a method previously described by Esumi *et al* in 1991 (13). Briefly, the cardiomyocytes were exposed to ischemia by replacing the medium with an ‘ischemic buffer’ designed to simulate the extracellular environment during myocardial ischemia, with the approximate concentrations of potassium, hydrogen and lactate ions that are observed to occur *in vivo* [137 mmol NaCl, 12 mmol KCl, 0.49 mmol MgCl_2_, 0.9 mmol CaCl_2_·2H_2_O, 4 mmol 4-(2-hydroxyethyl)-1-piperazineethanesulfonic acid (HEPES) and 20 mmol sodium lactate (pH 6.2)]. Cells were incubated in a hypoxic/ischemic chamber also known as the Modular Incubator Chamber (MIC-101; Billups-Rothenberg, Del Mar, CA, USA) at 37°C for 2 h in a humidified atmosphere of 5% CO_2_ and 95% nitrogen. For the reoxygenation process the cells were superfused in DMEM supplemented with 10% fetal calf serum at 37.1°C under 5% CO_2_ incubation for 2 h.

### Protein extraction and western blot analysis

B-cell lymphoma 2 (Bcl-2) and Bcl-2-associated X (Bax) protein concentrations were first determined using a bicinchoninic acid (BCA; Bio-Rad, Hercules, CA, USA) protein assay kit following the manufacturers’ instructions. Proteins were separated on a 10% sodium dodecyl sulfate polyacrylamide gel electrophoresis (SDS-PAGE) gel, transferred to a nitrocellulose membrane, blocked for 30 min at 37°C with 5% skimmed dry milk and incubated with a primary antibody overnight on a rocking platform. The primary antibodies used were the following: Mouse monoclonal against TLR4 (Abcam), rabbit against NFκB p50, rabbit against Bcl-2 and rabbit against Bax (all from Santa Cruz Biotechnology, Santa Cruz, CA, USA). Membranes were subsequently washed three times with Tris-buffered saline and Tween 20 (TBST) and incubated with the secondary antibody in TBST solution for 30 min at 37°C after which they were washed as above. The secondary antibodies used were the following: Peroxidase-labeled goat anti-rabbit IgG and peroxidase-labeled rabbit anti-mouse IgG (both from Zhongshan Company, Beijing, China). Immunoblots were developed using an enhanced chemiluminescent reagent kit (Abcam, Cambridge, UK). The bands were scanned and quantified by densitometric analysis using an image analyzer (Tanon2500, Shanghai, China).

### Flow-cytometric analysis

Dual staining with Annexin V-fluorescein isothiocyanate (FITC)/propidium iodide (PI) (Bestbio, Shanghai, China) was conducted to detect cell apoptosis. Flow cytometric analysis was performed 24 h after reperfusion. The procedures were carried out in accordance with the manufacturers’ instructions. Cells were harvested by trypsinization, washed twice with phosphate-buffered saline (PBS) and resuspended in binding buffer prior to the addition of Annexin V-FITC/PI. The mixture was incubated for 15 min in the dark at room temperature. Subsequently, cellular fluorescence was measured by bivariate flow cytometry using a FACScan system (BD Biosciences, Franklin Lakes, NJ, USA) and analyzed with CellQuest^™^ software (BD Biosciences). Annexin V-FITC/PI dual staining discriminated between intact cells (Annexin V^−^/PI^−^), apoptotic/early apoptotic cells (Annexin V^+^/PI^−^) and necrotic/late apoptotic cells (Annexin V^+^/PI^+^).

### Fluorescence quantitative polymerase chain reaction (qPCR)

The amounts of TLR4 and NF-κB were measured using fluorescence qPCR. At the end of each experiment, cells were collected and the total RNA was isolated using Gibco^®^ TRIzol^®^ reagent (Invitrogen Life Technologies, Carlsbad, CA, USA). The total RNA (8 μl) was reverse transcribed and 1 μl of the product was subjected to qPCR in the presence of specific primers. The sequences of the primers were as follows: TLR4 forward: 5′-TCATGCTTTCTCACGGCCTC-3′ and reverse: 5′-AGGAAGTACCTCTATGCAGGGAT-3′; NF-κB forward: 5′-ACGATCTGTTTCCCCTCATC-3′ and reverse: 5′-TGCTTCTCTCCCCAGGAATA-3′; β-actin: forward: 5′-CGCGAGTACAACCTTCTTGC-3′ and reverse: 5′-CGTCATCCATGGCGAACTGG-3′). The conditions for all qPCR reactions were optimized using an Applied Biosystems^®^ 7500 iCycler iQ system (Invitrogen Life Technologies) for a 20-μl reaction using the following 40-cycle program: 95°C for 10 min, 95°C for 15 sec and 60°C for 1 min. All samples were amplified simultaneously in triplicate in a one assay-run. In each reaction, β-actin was included as an internal standard and the relative quantitative gene expression was calculated using the 2^−ΔΔCt^ method.

### Statistical analysis

All values are expressed as mean ± standard deviation. The results were analyzed using analysis of variance (ANOVA) for multiple comparisons followed by two-sided Dunnett’s or Student-Newman-Keuls tests. P<0.05 was considered to indicate a statistically significant difference.

## Results

### Apoptosis rate of cardiomyocytes

**Figure 1:**
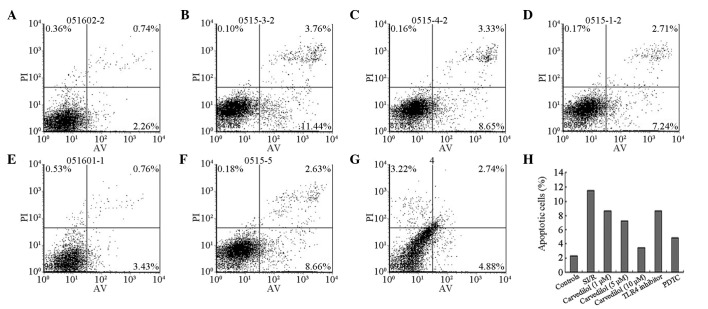
Flow-cytometric analysis was performed to detect the apoptotic rate of H9c2 cardiomyocytes under different treatments following simulated ischemia/reperfusion (SI/R) injury. The apoptosis rate was measured by Annexin V-fluorescein isothiocyanate (FITC)/propidium iodide (PI) dual staining cytometry. The upper right region shows the late apoptotic cells (FITC^+^/PI^+^); the lower left region shows the intact cells (FITC^−^/PI^−^) and; the lower right region shows the early apoptotic cells (FITC^+^/PI^−^). Data are presented as mean ± standard deviation. (A) Control; (B) SI/R; (C) carvedilol (1 μM); (D) carvedilol (5 μM); (E) carvedilol (10 μM); (F) toll-like receptor 4 (TLR4) inhibitor and; (G) pyrrolidine dithiocarbamate (PDTC; NF-κB inhibitor) groups. (H) Percentage of apoptotic cells in the various groups.^*^ P<0.01 compared with control group; ^**^ P<0.05 compared with SI/R group; ^#^P<0.01 compared with SI/R group.

Flow-cytometric analysis demonstrated that carvedilol exhibited anti-apoptotic effects (Fig. 1). Following 24 h of reperfusion, the cardiomyocyte apoptosis rate was markedly increased in the SI/R group when compared with that of the control group (P<0.01). The two lower concentrations of carvedilol (1 and 5 μM) decreased the apoptotic index to a similar extent, whereas the high dose (10 μM) had a much larger apoptosis-inhibiting effect when compared with the degree of apoptosis in the SI/R group (P<0.01). When the activation of TLR4 was inhibited by the TLR4 antibody and the activation of NF-κB was inhibited by PDTC, the apoptotic index of the H9c2 cells was significantly decreased compared with that of the SI/R group .

### Expression levels of B-cell lymphoma 2 (Bcl-2) and Bcl-2-associated X protein (Bax)

**Figure 2:**
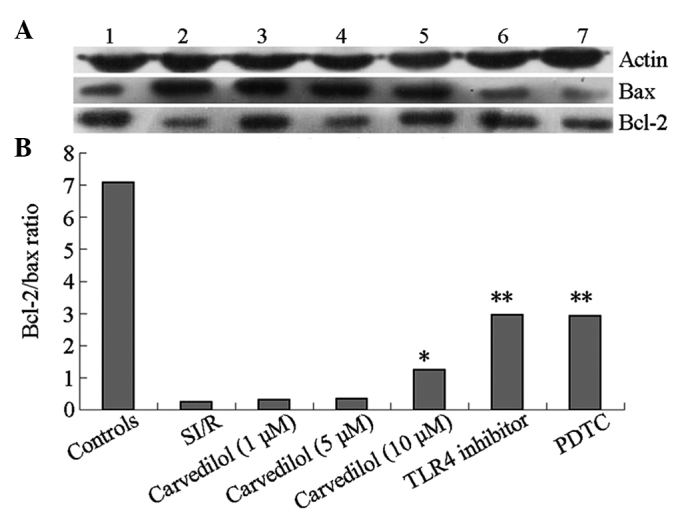
Effect of carvedilol on the expression levels of B-cell lymphoma 2 (Bcl-2) and bcl-2-associated X protein (Bax) in H9c2 cardiomyocytes under different treatments, following simulated ischemia/reperfusion (SI/R) injury. (A) Western blots of Bcl-2 and Bax and (B) the Bcl-2/Bax ratios in the various groups. Data are presented as the mean ± standard deviation. TLR4, toll-like receptor 4; PDTC, pyrrolidine dithiocarbamate. ^*^P<0.05 compared with the SI/R group; ^**^ P<0.01 compared with the SI/R group.

The Bcl-2/Bax ratio was decreased in the current SI/R injury model when compared with that of the control group (Fig. 2). Carvedilol at low and medium concentrations had no effect on the Bcl-2/Bax ratio during reperfusion. However, a high concentration of carvedilol resulted in a significant increase in the Bcl-2/Bax ratio compared with that in the SI/R group; the increase was paralleled by those observed in TLR4 inhibitor and NF-κB inhibitor groups, .

### TLR4 and NF-κB gene expression

**Figure 3:**
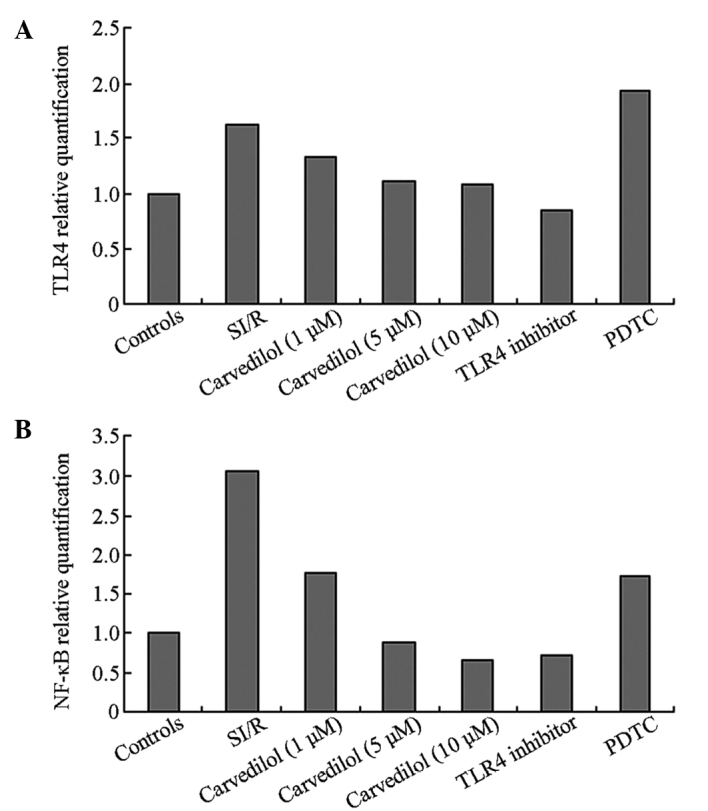
Effect of carvedilol on the expression levels of toll-like receptor 4 (TLR4) and nuclear factor κ-light-chain-enhancer of activated B cells (NF-κB) in H9c2 cardiomyocytes under different treatments, following simulated ischemia/reperfusion (SI/R) injury. PDTC, pyrrolidine dithiocarbamate. ^*^P<0.01 compared with control group; ^**^P<0.01 compared with SI/R group; ^#^P<0.01 compared with SI/R group.

The gene expression levels of TLR4 and NF-κB were upregulated in the SI/R model when compared with those in the control group (Fig. 3). Following the administration of carvedilol, the expression levels of TLR4 and NF-κB significantly decreased compared with those in the SI/R group. The highest concentration (10 μM) of carvedilol had the most pronounced effect on gene expression out of all the carvedilol treatments.

## Discussion

Carvedilol is widely used in the treatment of cardiac disease as a non-selective β-blocker. Previous studies have demonstrated that carvedilol reduces the risk of ischemic events following acute myocardial infarction (14). However, the particular pathways and underlying mechanisms involved in this process have yet to be elucidated. Apoptosis has been recognized as a component of a number of common cardiac pathologies, including ischemia (15), and the TLR4/NF-κB pathway is considered to be a major signal transduction pathway involved in cardiomyocyte apoptosis (16,17). In the present study, the cardioprotective effect of carvedilol was confirmed, especially at high concentrations, and it was shown that this effect is at least partially mediated by reductions in the expression levels of TLR4 and NF-κB.

Carvedilol is considered to be superior to other β-adrenoceptor blockers for the alleviation of heart failure at the clinical level; this is likely to be through its action as a regulator of apoptosis (18,19). The present study demonstrated that carvedilol significantly inhibited apoptosis following *in vitro* SI/R (2 h/24 h) in a concentration-dependent manner. The authors suggest that these dose-dependent anti-apoptotic effects of carvedilol should be taken into consideration in order to improve the prognosis of patients with I/R injury. However, further *in vivo* studies are required in order to determine the ideal doses.

Bcl-2 and Bax genes are widely used to evaluate cell survival/apoptosis following an apoptotic stimulus (20). Bax exhibits pro-apoptotic actions whereas Bcl-2 has an anti-apoptotic effect. Therefore, the ratio of Bax to Bcl-2 is an effective predictor of the apoptotic fate of a cell (21). As predicted, in the current study the Bcl-2/Bax ratio decreased during SI/R-induced apoptosis (P<0.01, compared with the control group) and this effect was blocked following the administration of carvedilol. Furthermore, the present study revealed that a high concentration of carvedilol (10 μM) had the largest effect on the ratio of Bcl-2/Bax. These data suggest that high dosages of carvedilol are able to significantly suppress the apoptotic effects of SI/R by blocking regulatory proteins. Once again, the clinical implications of using higher doses of carvedilol must be further studied in order to clarify the effects of this cardioprotective drug.

The current study also investigated the mechanisms that are potentially involved in the observed anti-apoptotic effect of carvedilol. TLRs are a family of molecules that play a critical role in the regulation of innate immunity, which is a significant component of myocardial I/R (MI/R) injury. When inhibited, TLRs protect against MI/R-induced damage in the heart (3,22). Therefore, the authors hypothesized that the anti-apoptotic action of carvedilol may be associated with a TLR4/NF-κB-mediated response. The present study demonstrated that the expression levels of TLR4 and NF-κB increased during SI/R. Furthermore, the expression level of NF-κB decreased significantly following the inhibition of TLR4 by the TLR4 antibody. These results were verified by fluorescence qPCR. The results indicate that carvedilol-mediated inhibition of apoptosis is regulated, to a significant extent, through the TLR4/NF-κB pathway.

In conclusion, the present study demonstrated that carvedilol inhibits SI/R-induced apoptosis *in vitro* in cardiomyocytes via the TLR4/NF-κB-mediated pathway. These results may be important in elucidating certain key factors involved in the molecular mechanisms of the cardioprotective capabilities of carvedilol.

## References

[1] Jayachandran M, Brunn GJ, Karnicki K, Miller RS, Owen WG, Miller VM (2007). *In vivo* effects of lipopolysaccharide and TLR4 on platelet production and activity: implications for thrombotic risk. J Appl Physiol (1985).

[2] Zhong X, Li X, Qian L (2012). Glycine attenuates myocardial ischemia-reperfusion injury by inhibiting myocardial apoptosis in rats. J Biomed Res.

[3] Lin J, Wang H, Li J (2013). κ-Opioid receptor stimulation modulates TLR4/NF-κB signaling in the rat heart subjected to ischemia-reperfusion. Cytokine.

[4] Kim SC, Stice JP, Chen L (2009). Extracellular heat shock protein 60, cardiac myocytes, and apoptosis. Circ Res.

[5] Zhao P, Wang J, He L (2009). Deficiency in TLR4 signal transduction ameliorates cardiac injury and cardiomyocyte contractile dysfunction during ischemia. J Cell Mol Med.

[6] Riad A, Jäger S, Sobirey M (2008). Toll-like receptor-4 modulates survival by induction of left ventricular remodeling after myocardial infarction in mice. J Immunol.

[7] Leonetti G, Egan CG (2012). Use of carvedilol in hypertension: an update. Vasc Health Risk Manag.

[8] Kanoupakis EM, Manios EG, Mavrakis HE (2008). Electrophysiological effects of carvedilol administration in patients with dilated cardiomyopathy. Cardiovasc Drugs Ther.

[9] Le DE, Pascotto M, Leong-Poi H, Sari I, Micari A, Kaul S (2013). Anti-inflammatory and pro-angiogenic effects of beta blockers in a canine model of chronic ischemic cardiomyopathy: comparison between carvedilol and metoprolol. Basic Res Cardiol.

[10] Mori Y, Nishikawa Y, Kobayashi F, Hiramatsu K (2013). Clinical status and outcome of Japanese heart failure patients with reduced or preserved ejection fraction treated with carvedilol. Int Heart J.

[11] Chen-Scarabelli C, Saravolatz L, Murad Y (2012). A critical review of the use of carvedilol in ischemic heart disease. Am J Cardiovasc Drugs.

[12] Liu Q, Zhang J, Xu Y, Huang Y, Wu C (2013). Effect of carvedilol on cardiomyocyte apoptosis in a rat model of myocardial infarction: a role for toll-like receptor 4. Indian J Pharmacol.

[13] Esumi K, Nishida M, Shaw D, Smith TW, Marsh JD (1991). NADH measurements in adult rat myocytes during simulated ischemia. Am J Physiol.

[14] Dargie HJ (2001). Effect of carvedilol on outcome after myocardial infarction in patients with left-ventricular dysfunction: the CAPRICORN randomised trial. Lancet.

[15] Jin YC, Kim CW, Kim YM (2009). Cryptotanshinone, a lipophilic compound of *Salvia miltiorrriza* root, inhibits TNF-alpha-induced expression of adhesion molecules in HUVEC and attenuates rat myocardial ischemia/reperfusion injury *in vivo*. Eur J Pharmacol.

[16] Lin E, Freedman JE, Beaulieu LM (2009). Innate immunity and toll-like receptor antagonists: a potential role in the treatment of cardiovascular diseases. Cardiovasc Ther.

[17] Ishikawa Y, Satoh M, Itoh T, Minami Y, Takahashi Y, Akamura M (2008). Local expression of Toll-like receptor 4 at the site of ruptured plaques in patients with acute myocardial infarction. Clin Sci (Lond).

[18] Poole-Wilson PA, Swedberg K, Cleland JG (2003). Carvedilol Or Metoprolol European Trial Investigators: Comparison of carvedilol and metoprolol on clinical outcomes in patients with chronic heart failure in the Carvedilol Or Metoprolol European Trial (COMET): randomised controlled trial. Lancet.

[19] Fiuzat M, Wojdyla D, Kitzman D (2012). Relationship of beta-blocker dose with outcomes in ambulatory heart failure patients with systolic dysfunction: results from the HF-ACTION (Heart Failure: A Controlled Trial Investigating Outcomes of Exercise Training) trial. J Am Coll Cardiol.

[20] Soriano ME, Scorrano L (2011). Traveling Bax and forth from mitochondria to control apoptosis. Cell.

[21] Condorelli G, Morisco C, Stassi G (1999). Increased cardiomyocyte apoptosis and changes in proapoptotic and antiapoptotic genes bax and bcl-2 during left ventricular adaptations to chronic pressure overload in the rat. Circulation.

[22] Shimamoto A, Chong AJ, Yada M (2006). Inhibition of Toll-like receptor 4 with eritoran attenuates myocardial ischemia-reperfusion injury. Circulation.

